# The Use and Impact of Cognitive Enhancers among University Students: A Systematic Review

**DOI:** 10.3390/brainsci11030355

**Published:** 2021-03-10

**Authors:** Safia Sharif, Amira Guirguis, Suzanne Fergus, Fabrizio Schifano

**Affiliations:** 1 Psychopharmacology, Substance Misuse and Novel Psychoactive Substances Research Unit, School of Life and Medical Sciences, University of Hertfordshire, Hatfield AL10 9AB, UK; s.sharif2@herts.ac.uk (S.S.); f.schifano@herts.ac.uk (F.S.); 2 Institute of Life Sciences 2, Swansea University, Swansea SA2 8PP, Wales, UK

**Keywords:** neuroenhancement, cognitive enhancement, drug abuse, university students, study drugs, non-medical drug use, smart drugs

## Abstract

Introduction: Cognitive enhancers (CEs), also known as “smart drugs”, “study aids” or “nootropics” are a cause of concern. Recent research studies investigated the use of CEs being taken as study aids by university students. This manuscript provides an overview of popular CEs, focusing on a range of drugs/substances (e.g., prescription CEs including amphetamine salt mixtures, methylphenidate, modafinil and piracetam; and non-prescription CEs including caffeine, cobalamin (vitamin B12), guarana, pyridoxine (vitamin B6) and vinpocetine) that have emerged as being misused. The diverted non-prescription use of these molecules and the related potential for dependence and/or addiction is being reported. It has been demonstrated that healthy students (i.e., those without any diagnosed mental disorders) are increasingly using drugs such as methylphenidate, a mixture of dextroamphetamine/amphetamine, and modafinil, for the purpose of increasing their alertness, concentration or memory. Aim: To investigate the level of knowledge, perception and impact of the use of a range of CEs within Higher Education Institutions. Methodology: A systematic review was conducted in adherence with the Preferred Reporting Items for Systematic Reviews and Meta-Analyses (PRISMA) guidelines. Whilst 1400 studies were identified within this study through a variety of electronic databases (e.g., 520 through PubMed, 490 through Science Direct and 390 through Scopus), 48 papers were deemed relevant and were included in this review. Results: The most popular molecules identified here included the stimulant CEs, e.g., methylphenidate, modafinil, amphetamine salt mixtures and caffeine-related compounds; stimulant CEs’ intake was more prevalent among males than females; drugs were largely obtained from friends and family, as well as via the Internet. It is therefore suggested that CEs are increasingly being used among healthy individuals, mainly students without any diagnosed cognitive disorders, to increase their alertness, concentration, or memory, in the belief that these CEs will improve their performance during examinations or when studying. The impact of stimulant CEs may include tolerance, dependence and/or somatic (e.g., cardiovascular; neurological) complications. Discussion: The availability of CEs for non-medical indications in different countries is influenced by a range of factors including legal, social and ethical factors. Considering the risk factors and motivations that encourage university students to use CE drugs, it is essential to raise awareness about CE-related harms, counteract myths regarding “safe” CE use and address cognitive enhancement in an early stage during education as a preventative public health measure.

## 1. Introduction

Cognitive enhancement is defined as an “amplification or extension of core capacity of the mind by improving the internal and external information processing systems” [1]. Cognitive enhancement can be achieved in two ways, e.g., “pharmacologically”, by taking cognitive enhancer (CE) drugs/substances; or “non-pharmacologically”, by maintaining a healthy lifestyle, which includes being physically, mentally and socially active; eating a healthy, balanced diet; drinking alcohol only in moderation; and maintaining good sleep habits [2]. CEs, also known as “smart drugs” or “nootropics”, are a heterogeneous group of chemical substances that are used to improve cognitive function [3], particularly memory, alertness, attention, learning performance, creativity and motivation [4]. CEs are typically being obtained, and at times by healthy individuals [5,6], on prescription, over-the-counter, online, or through other sources such as family or friends [7]. The clinical impact of CEs’ ingestion can be significant, with these molecules being able to affect various neurotransmitter pathways in the brain, including the cholinergic, dopaminergic, noradrenergic and serotonergic pathways [8]. Whilst their mechanism of action is not fully understood [3], most popular CEs (e.g., methylphenidate, modafinil and amphetamine salt mixtures) are stimulants [9]. Methylphenidate increases the levels of noradrenaline (NA) and dopamine (DA) in both the prefrontal cortex and the cortical/subcortical regions, and this effect may be associated with levels of improved attention in Attention Deficit Hyperactivity Disorder (ADHD) [10]. Conversely, with modafinil—a medicine being used to treat narcolepsy—stimulant actions are associated with an impact on NA, glutamate (NMDA or N-methyl-D-aspartate) and DA [11]. In particular, modafinil increases DA levels in the caudate and nucleus accumbens (Nac), whilst blocking DA transporters in a healthy individual’s brain [12]. Out of these molecules, modafinil may be better tolerated, inducing less adverse drug reactions, whilst not being associated with a high risk of dependence [13]. The amphetamine salt mixtures (e.g., in the branded product Adderall) block the re-uptake of both NA and DA into the pre-synaptic neuron, and increase their release as well from the pre-synaptic neuron, hence increasing their concentrations in the synaptic cleft [14].

Indeed, since the 1940s, both modafinil and amphetamine (e.g., “go pills”) CE categories have been the subject of military research, to help soldiers stay alert whilst attenuating the effects of sleep deprivation [15,16]. However, these drugs are increasingly being used by healthy individuals, including students and night shift workers, to improve their cognitive and motivational functions [17]. Associations between CEs and drugs in sports have been investigated [18]. CEs and drugs in sports share many aspects with respect to “enhancement” and “doping”. The former may be more socially acceptable, whilst the latter is considered illegal and is heavily monitored by organisations such as The World Anti-Doping Agency (WADA). The use of freely available CEs, such as caffeinated products and vitamins, have been investigated in athletes as “gateway” and “predictor” of physical enhancers use. The non-monitored CEs were found to be highly used among athletes with or without physical enhancer use [19]. Studies also showed that users of erogonomic aids such as caffeine may favour doping due to “biased reasoning patterns” [19].

It is important to note that students using CEs do not only aim to achieve a cognitive enhancement, but also a motivational enhancement and an overflow of energy. They may use a combination of CEs as well as alcohol, and/or recreational sedatives, in an attempt to achieve a good quality sleep, reduce nervousness and improve overall performance in exams and study-related assessments [20,21].

The lifetime prevalence rate of prescribed CEs’ intake for non-medical reasons, as a self-attempt to increase cognitive performances, among university students in the UK and Ireland has been estimated to be around 10% [22]. However, these levels of intake may be underestimated [23] and the trend has attracted a considerable interest [22], relating to its social, ethical and legal implications [24,25,26]. Whilst most studies have focused on the prevalence of a limited range of a few CEs (e.g., amphetamine salt mixtures, methylphenidate and modafinil), focusing on intake by students in Higher Education Institutions (HEIs), a study by Napoletano et al. (2020) identified a total of 142 unique CEs. These molecules were then sub-grouped into 10 categories, according to recently proposed classifications [27] including: prescribed drugs, plants/herbs/products, psychostimulants; image- and performance-enhancing drugs (IPEDs), miscellaneous, GABAergic (gamma- aminobutyric acid-ergic) drugs, phenethylamines, cannabimimetics, tryptamine derivatives, and piperazine derivatives. In parallel with the continuous emergence of new/novel psychoactive substances (NPS), which has enriched the repertoire of illicit drug use [28], this manuscript aims to provide an updated overview of the use of CEs among university students.

## 2. Methods

The current systematic literature review was performed in adherence with the Preferred Reporting Items for Systematic Reviews and Meta-Analyses (PRISMA) guidelines [29], to estimate CEs’ prevalence of intake; and assess knowledge, awareness and impact of CEs’ use among university students.

### 2.1. Literature Search (Inclusion and Exclusion Criteria)

The focus here was on quantitative and qualitative studies relating to CEs’ use among University students: The literature search was performed using a range of key word strings, e.g., cognitive enhancers AND neuroenhancement, prescription drug misuse OR prescription drug abuse among healthy individuals AND enhancement. In particular, the search strategy was conducted from three databases Scopus, PubMed and Science direct. Finally, a manual search was also carried out using Google Scholar in order to ensure none of the key articles and studies were missed.

Inclusion criteria were quantitative (surveys) and qualitative (interviews) studies having been carried out among healthy students aged 18 years and older in HEIs. Articles were included if they related to a range of nine CEs (prescription CEs including amphetamine salt mixtures, methylphenidate, modafinil and piracetam; and non-prescription CEs including caffeine, cobalamin (vitamin B12), guarana, pyridoxine (vitamin B6) and vinpocetine), which were selected here because of their popularity among university students [4,7,22,26,30]. Studies written in English, from the year 2000 (i.e., from around the time when NPS started to emerge in drug scenarios) to 2020 were included in the study search. Regional/world drug reports (e.g., from the European Monitoring Centre for Drugs and Drug Addiction/EMCDDA and the United Nations Office for Drug and Crime/UNODC) were included here as well. Conversely, studies focussing on underage children, on preclinical experiments or students with medical diagnoses using the selected drugs/substances for medical reasons were excluded from the study. Non-English articles were also excluded.

### 2.2. Quality Assessment

Based on the inclusion criteria, the selected articles were appraised for quality using PRISMA checklists [29]. Search results were exported to Mendeley, a free reference manager and academic social network. This tool was used to determine the structure of the index study methodology [29].

## 3. Results

### 3.1. Summary of the Literature Search

**Figure 1:**
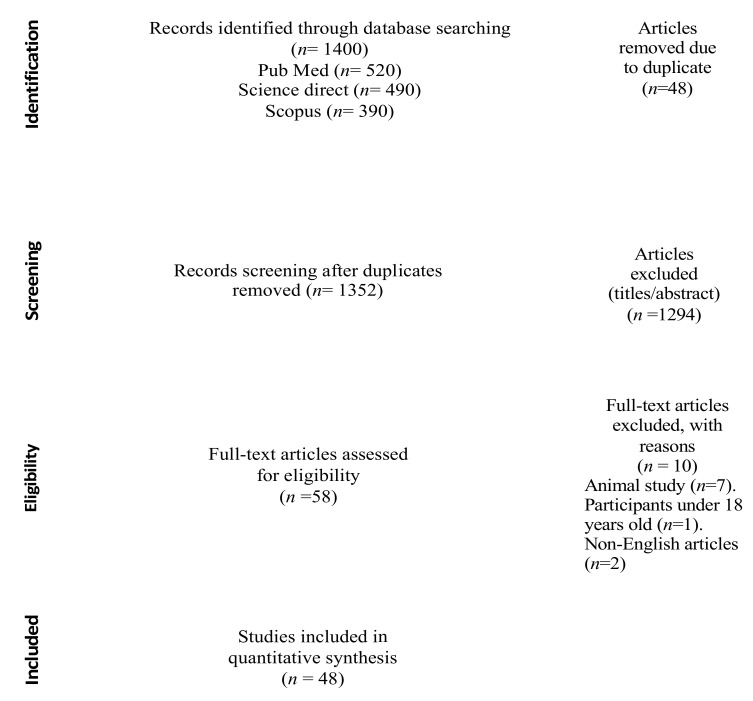
Cognitive enhancers’ intake by university students: Preferred Reporting Items for Systematic Reviews and Meta-Analyses (PRISMA) Flow Diagram.

The literature search identified a total of 1400 studies here (e.g., 520 through PubMed, 490 through Science Direct and 390 through Scopus) (Figure 1). Forty-eight studies were excluded as they were duplicates, 1294 studies were screened and were excluded based on their title and abstract, 10 did not meet the inclusion criteria, and 48 were deemed relevant and were included in this review (Table 1).

Table 1 shows the summary of findings from the literature review on the prevalence of CEs among university students.

Nine studies were conducted in the UK (i.e., six survey studies, two interviews and one mixed methods study). The remaining studies included survey studies that were conducted in the USA (*n* = 8) and Iran (*n* = 4). In Australia, three surveys and one interview, in Canada, two surveys and one focus group interview, in Germany, three surveys and one interview were conducted. Three survey studies were carried out in each of the following countries: Brazil, France, Italy and Switzerland. Two survey studies were carried out in each of the following countries: Austria, Belgium, Greece, New Zeeland and the Netherlands; and one survey study was carried out in each of the following countries: Hungary, Iceland, Ireland, Lithuania, Pakistan, Portugal, South Africa and UAE. Finally, one mixed-methods study was carried out in both Lithuania and the Netherlands. Participants were students from a range of disciplines, including Medicine, Pharmacy, Engineering, Law, Computer Science, Business, Education, Psychology and Social Sciences. The sample size of the different studies ranged between 77 and 80,000 participants each.

An overview of the demographic variables, prevalence of use, technical knowledge of CEs, motivations for use, source of CEs’ acquisition and positive/negative subjective effects is summarised here.

1. Demographics’ variables

Males were here identified as the most typical CE misusers [7,22,30,31,41,51,52,63,66,70,71,73,74,75], with some studies reporting a male:female ratio of 3:1 [54]. In contrast with this, a Welsh study reported that female representation was slightly more than males [68].

2. Prevalence of CEs’ use

A growth of CEs’ intake over the past few years has been reported, including from both high-ranking universities and highly competitive courses such as Medicine and Pharmacy [41]. In the UK, findings showed that 33% of the participants used CEs which were not prescribed to them for the purpose of study [68]. In a survey conducted among UK and Ireland university students, it was found that the lifetime prevalence of the use of modafinil, methylphenidate and amphetamine were, respectively, 6.2%, 5.9%, and 2% [22]. Conversely, the lifetime prevalence of CEs’ intake among University students in the US was estimated to range between 5% and 43% [76]. More precisely, a meta-analysis from the US estimated that the misuse of CEs among university students was 17% [75]. Compared to the US, most British university students may be more cautious in using prescription drugs as CEs [49].

A recent study in Brazil reported that, out of 1865 students from different academic disciplines, 4.2% reported to having had used CEs in the last 12 months, with the most popular molecule having been methylphenidate which was not associated with an ADHD diagnosis. With respect to what is being described in less competitive study fields [77], Medicine and Pharmacy have been identified as being both stressful and highly competitive academic courses worldwide [44,45]. In this respect, a study that was conducted among medical students in Iran (2000–2007) showed that methylphenidate users’ mean knowledge score was higher than that of non-users (*p* = 0.008), with age (range 18–28 years), sex (male 92.5%) and 26% fourth school year having been positively correlated with knowledge score (*p* < 0.05). Some 8.7% of participants had taken methylphenidate at least once in their lifetime [70]. Similarly, a study carried out in Lithuania reported that the point prevalence of CEs (modafinil, methylphenidate and amphetamine) among medical students was 8.1% [77].

Finally, caffeine use as a CE has grown in popularity worldwide [78]. A study in the UAE assessed the prevalence and perceived benefits of caffeinated beverage consumption among university students [51]. More than 98.5% of the study participants were shown to be caffeine consumers, with 31% having reported being addicted to caffeine; heavy caffeine consumption was significantly associated with heart problems [51].

Despite the global popularity of the non-prescription caffeine, most research articles report the use of prescription CEs among university students. Therefore, the true prevalence of prescription vs. non-prescription CEs among university students is not fully understood and, hence, more research is needed.

### 3.2. CEs’ Knowledge and Reported Positive/Negative Effects

University students may be attracted by stimulant drugs for several reasons, e.g., to increase awake time, enhance cognitive performance, improve professional and academic achievement [41], but also to help with socialising and getting high [79]. Indeed, the main motivations for misusing methylphenidate may relate to improving concentration (65.2%), helping with studying (59.8%) and increasing wakefulness (47.5%) [73]. Other studies have associated methylphenidate misuse with the need to help with concentration, stay alert, have more energy and improve self-confidence levels [17,34,70]. A 2019 UK qualitative study with Biomedical Science undergraduate students examined their understanding of the risks of non-prescribed drugs, and particularly modafinil, misuse. Drivers of use were related to university pressures and desires to increase productivity; the customisation of the sleep–wake cycle was described as a key benefit of ‘study drug’ use [32].

Increasing the levels of cognitive performance may indeed potentially allow students to study for more hours, and/or increase working memory performance [80]. According to Greely et al. (2008), modafinil may be chosen as a CE because of its high online accessibility and availability. Conversely, whilst studies in the UK suggested that CE drugs such as modafinil can enhance thinking skills [81], over-confidence was reported as one of the CE’s side effects, together with a high risk of dependence [2].

The popularity of caffeine and related products as CEs may be related to the need to boost energy, stay awake, improve mood, increase concentration and socialise [51]. In the UAE, the mean level of knowledge about caffeine was described as less than 33%. Younger participants (*p* = 0.008) and those who worked in healthcare and education (*p* < 0.001) were significantly more knowledgeable about its negative effects, including anxiety, insomnia, tachycardia, irritability and muscle tremors [51]. A recent systematic review focussing on the effects of the caffeine-containing plant Paullinia cupana (“guarana”) on cognition in young, healthy adults found improved levels in both reaction time and accuracy performance [82]. Guarana has also been described to improve memory performance and increase alertness levels [83]. Long-term use of high dose of guarana can, however, result in a series of adverse effects, including irritability, palpitation and anxiety [2,84].

Despite the legal restrictions that control the possession and supply of controlled CEs, students often obtain them due to their desired pharmacological effects. Table 2 summarises the desired effects of CEs, their neuro-modulatory effects and their legal classification.

Mixtures of CE substances/drugs used by healthy students to improve cognition is on the rise and is being considered as a type of “academic doping” [85]. Poly-CE use has been documented in previous studies [86]. In Switzerland, users reported ingesting methylphenidate in addition to other CEs. Others reported using both modafinil in addition to Alzheimer’s disease drugs. Others ingested antidepressants in combination with Parkinson’s disease drugs [86]. Studies have shown that methylphenidate users were more likely to use illicit substances as well e.g., marijuana and ecstasy (MDMA or 3,4-methylene dioxymethamphetamine) as compared to other prescription CE users [9].

Poly-CE use with psychostimulant and other effects offers both synergistic and additive effects based on used substances, hence potentially combining cognitive effects with wakefulness; emotional and/or motivational effects; mood-, performance-, and executive functioning-enhancing and euphoric effects [87,88], with risks to health that may range from mild to serious risks including dependence, tolerance and neurological, psychological and cardiovascular disorders, with a risk of overdose potentially leading to death.

The 2015 Western Australian Stimulant Regulatory Scheme showed that students may use CE to cope with study-related stress [89]. They also found that CE users are also regular illicit psychostimulant users, yet the relationship between CE and other psychostimulant such as MDMA (ecstasy) co-use/consumption is to be determined [90].

### 3.3. Sources of CEs’ Acquisition

Sources of CE acquisition may relate to friends and family [7,59]. Students diagnosed with ADHD, but not taking their methylphenidate medication regularly, have been reported as the main source for fellow students [4]. In another study, 75.5% of methylphenidate was identified as having been purchased from friends at a university campus whilst 64.3% of modafinil was obtained online [22]. Accessing the web for drug acquisition activities is a reason for concern [30], with young people (18–25 years old) being at high risk because there is no way to know what the actual ingredients of the drugs/substances are in those products [30], they are extensive users of the Internet [112]; it was found that over a third of the websites selling modafinil specifically recommended use of the drug to aid studying [30].

## 4. Discussion

The current systematic review provided an in-depth and updated understanding on CEs’ prevalence of use; levels of knowledge; and their impact on HEI university students, which is clearly a critical public health issue. The past few years have seen increasing levels of concern about the use of pharmaceutical cognitive enhancement among university students worldwide, with the lifetime prevalence of CEs misuse among these subjects ranging from 6% to 20%, depending on the study subject [67]. Of particular concern, however, is CE’s use in Health Sciences/ Biomedical students [34,36,38,39,45,48,54,57,63,67,70,71]. Most data initially emerged from the United States [61,75,113], eventually followed by reports from the United Kingdom [22], Australia [46,50]; and Europe, namely from France [53,60], and Italy [38,69].

The most popular prescription CEs among the selected ones in this study were modafinil, methylphenidate and amphetamine salt mixtures [71], with methylphenidate being the most popular among students [9]. Conversely, the most popular freely available CE was Caffeine [78]. Although not confirmed by some studies [113,114], males were identified here as more likely to use CE drugs than females [31,52,63,71,75,77]. Some studies also showed that, despite that the number of female participants was higher than their male counterparts, the rate of CE use was much higher than in female students [77].

Although no differences between genders in favouring methylphenidate as the most popular CE or in the preferential choice of any CE were recorded, there were gender differences in motivation for use [33]. Female students’ motivation for CE use were to increase concentration, memory, alertness and academic performance, and because “friends use it”. In contrast, male students’ motivations for CE use were mainly to increase study time and experiment [33].

In general, with regard to illicit substance use, Dr Adam Winstock (CEO of the Global drug Survey) pointed out the gender differences, explaining it as possibly resulting from societal stigma, shame and cultural expectations around women taking drugs. Additional factors that influence women’s decisions in using drugs include pregnancy and motherhood. Economic status and the lower rate of criminal activity amongst women also reduce female drug use and exposure to illicit drugs as compared to males [115].

Indeed, several social factors have been identified here to influence CEs’ use practices among university students [56]. These included: peer-pressure, competition, performance demands and prior drug use [85], but also recreation [79].

The availability of CEs for non-medical indications in the different countries is affected by a range of factors, including legal, social, and ethical factors [33,40,116]. Indeed, some CEs are being openly made available online [30,117], where they are marketed as “smart drugs”, “study drugs”, “plant food”, “research chemicals” and “designer drugs” as well [30,112]. The unregulated online access, and especially so for modafinil and methylphenidate, is likely to be associated with an increase in CEs’ non-medical use and subsequent harm [30]. Indeed, high levels of modafinil may have reportedly been sold and shipped to students at high-ranking/top UK universities, mostly during the examination period [118]. Conversely, as CEs’ legal alternative to either prescribing or illicit drugs of abuse, guarana was found here to be popular, with affordable online prices encouraging young users/students to buy greater quantities in order to receive discounts and free shipping [30,119].

Sahakian et al. (2008) opened a debate on the positive impact on improving cognitive functions, suggesting that benefits of CEs should be maximised, and their harm minimised [25]. In some studies, CE drugs have been shown to moderately enhance cognitive performance in healthy individuals [120]. Accordingly, CE tools including pharmacological cognitive enhancement could improve the quality of life of both busy workers and exhausted students to extend their work/academic productivity levels [121], hence benefitting both the individual and society [25]. There have been extensive reports focussing on CEs’ intake to aid concentration and memory among healthy individuals, including students, academics, shift workers, and even chess players to improve their cognitive performance [122]. A study by Smith and Farah (2011a) suggested that the effects of both methylphenidate and amphetamine salt mixtures on cognitive performances in healthy participants showed positively consistent effects in learning, but especially so in delayed recall and recognition testing, pointing to an effect on memory consolidation [4]. An additional study by Schelle et al. (2015) showed a positive effect of methylphenidate on memory and planning performance in healthy individuals. However, others have suggested that evidence regarding the clinical benefits of CEs in healthy individuals is still inconclusive [123]. A 2010 systematic review and meta-analysis of published randomised controlled trials of the effect of both modafinil and methylphenidate in healthy individuals showed that the anticipated effects of these two agents as cognitive enhancers exceeded their actual effect [80]. Hence, it has been suggested that the ability of amphetamine-type substance mixtures to enhance academic performance among students could be attributed to their effect on energy, confidence and motivation levels rather than to a direct effect on cognitive performances [124]. In fact, individuals may be biased in predicting their own performance, e.g., they either underestimate or overestimate their academic competence [125]. Moreover, cognitive improvement seems to vary considerably from one agent to another, and Smith et al. (2011) reported that one third of studies from past literature reviews showed null results. One could then argue that there are more unpublished studies in the literature with null results, due to publication bias favouring positive results [4].

On the other hand, use of stimulant CEs may be associated with negative academic performances in terms of the euphoric state induced, with abnormal mood elation preventing the student from spending enough time in preparation for an exam [17,57]. Furthermore, methylphenidate is reported to present with a dependence potential [126], and modafinil dependence cases have been identified as well [127]. It is also worth noting the amphetamine-type substance-related dependence; withdrawal; and psychosis issues [28]. Untoward effects relating to the index CE may indeed influence students’ choices, with them being keen to consider modafinil as opposed to methylphenidate and amphetamine salt mixtures. Indeed, Steward and Pickersgill (2019) found that all their CE users had ingested modafinil, with only some also having tried methylphenidate and amphetamine salt mixtures for the purpose of study. In fact, students described how the use of methylphenidate and amphetamine salt mixtures could result in dependence and hence these were approached more cautiously [32]. Overall, however, the use of methylphenidate has significantly increased, with its consumption, in defined daily doses, having increased to approximately 2.4 billion worldwide [35]. In the UK, both methylphenidate and amphetamine compounds are Class B controlled drugs [128]. This means they can be provided via prescription, but the maximum quantity issued should not exceed 30 days (unless justified by the prescriber) and a personal import/export licence is required to transport the drug in or out of the UK if the amount exceeds a 3-month supply [30]. Modafinil is a prescription-only medicine in the UK, but it is not controlled under the Misuse of Drugs Act 1971 or subject to scheduling under the Misuse of Drugs Regulations 2001; hence, it is illegal to supply it without a prescription, but it is not illegal to possess the drug for personal use [128]. To cope with these restrictions, CEs’ selling websites provide discreet packaging; offer free reshipment if the package is seized; and encourage third-party, difficult to track, payment methods [30]. This outcome suggests running campaigns that mitigate harm and raise awareness among students who use CE drugs. Finally, although caffeine is also a stimulant, its use is not associated with either acquisition, affordability, availability, or legality issues [42,66]. However, with caffeine high-dosage intake a range of medical and psychiatric effects can be observed, most typically including anxiety, panic attacks, sleeping disorders and cardiovascular issues [129].

A Cochrane review found no evidence that short-term intake of vitamins B6 and B12 supplements improve cognitive function or mood. The review did find some evidence that daily vitamin B6 and B12 supplements can affect biochemical indices of vitamin B6 and B12 status in healthy individuals, but these changes had no overall impact on cognition [107].

According to the review of the literature, the drugs selected were chosen based on their popularity among healthy university students, but the drugs most used among students were (modafinil, methylphenidate and Adderall) and, in terms of substances, caffeine was the most popular among university students. However, a study by Carlier, J (2019) reported that methylphenidate is one of the most popular CEs and several analogues appeared on the drug market during the last years. However, little or no scientific data on these new analogues are available.

As sports organisations such as WADA are overviewing and prohibiting the use of physical enhancers, no such control exists in schools and universities. Therefore, in order to decrease the long-term deleterious effects of CEs in individuals who use them, government-level interventions are urgently required.

A harm reduction programme is also recommended to reduce the negative, legal and societal impact of substance use [125]. The programme should consider supporting individuals with problematic substance use and their families with compassion and appropriate advice and interventions, whilst safeguarding their dignity [125]. These findings suggest the importance of raising awareness of the harms of CE use, provide accurate knowledge, counteract myths regarding “safe” CE use and address cognitive enhancement in an early stage during education as a preventative public health measure.

## 5. Limitations

There are a few limitations that were considered in this manuscript. The first limitation is related here to the sole focus on English language studies having been included in the search; future studies should consider further languages. The second limitation relates to the methods used by the different studies, typically involving self-reporting surveys which could have introduced biases. Finally, the current study focused only on undergraduate students; however, postgraduate students, academic staff, and remaining workers should be considered by future studies.

## 6. Conclusions

A number of students worldwide may be willing to consider CEs’ ingestion to improve their academic performances. The attitude of university students about CEs and their possible benefits is, however, based on anecdotal, and arguably biased, information obtained from the media, the web, and friends [130]. Overall, it seems from this review that the topic is not being sufficiently covered in the curriculum of modern universities. Conversely, this issue should be discussed, as an inter-professional or inter-disciplinary learning opportunity, from a public health perspective [7,44]. CEs’ use may arguably be reduced if students’ levels of awareness were raised, emphasising that CEs’ intake may pose a risk to safety, and especially so in vulnerable individuals [31]. Indeed, impacts of CE drugs’ intake may include tolerance, dependence, withdrawal, cardiovascular and neurological disorders with a related risk of death due to overdose [28,117,131]. The implementation of a harm reduction campaign, in order to bring the overall consumption down, has been proposed as well [41,91].

Finally, Shaw (2014) suggested that one of the most fascinating issues in the emerging field of neuroethics is pharmaceutical cognitive enhancement. Medical debate [21,132] has largely focused on the CEs’ potential to help those who are cognitively impaired. Hence, it is here suggested that CEs’ use by university students, seems to raise the issue of “cosmetic” neuropsychopharmacology [133,134].

## Figures and Tables

**Table 1:** Summary of the literature review focusing on Cognitive Enhancer (CE) drug(s)/substance(s) being considered for the study (e.g., methylphenidate, amphetamine, modafinil and piracetam, caffeine pills, guarana, cobalamin (vitamin B12), vinpocetine and pyridoxine (vitamin B6).

Reference	Country	Prescription CE	Non-Prescription CE	Study Sample	Methodology Used	Results/Lifetime Prevalence of CE	Notes/Limitations
[30]	UK	Ritalin, Dextroamphetamine/amphetamine, Modafinil and Armodafinil	-	Survey = 506 participants. Interview = 15 participants	Online survey and Interview.	Out of 506 participants, 45.5% (*n* = 230) reported that they had used a range of CE drugs in the previous 12 months for the purpose of study. Male usage was reported as being more than two and a half times higher than female usage.	The motivational factors behind CE use were investigated with greater understanding of the factors influencing their use. Universities need to develop a greater awareness of the prevalence of CE use amongst their students and consider taking an active approach in reducing their use.
[7]	Brazil	Modafinil Methylphenidate Piracetam	-	1865 participants.	Online survey.	Out of 1865 respondents, 4.2% had used CEs in the previous 12 months, and the prevalence among Law students reached 14.3%. The most commonly used smart drug was methylphenidate. The drug was mostly obtained through a friend.	The limitation was the questionnaire itself. It is possible that there was a memory bias and omission of response, underestimating the prevalence found. However, the limitation was reduced as the questionnaire was self-administered and anonymous.
[31]	UK	Modafinil Amphetamine Methylphenidate Beta Blockers	-	612 participants from Russell Group universities.	Online survey.	17% reported having used smart drugs previously.	The limitation of this study was that it was open to all UK full-time undergraduate students, although the majority of the participants were from Russell Group universities. However, they did not collect additional data on discipline or university and, therefore, cannot be sure how representative the sample is for the UK university population as a whole or the population at the host university. This means that factors such as competitiveness cannot be extracted from the data.
[32]	UK	Modafinil	-	15 undergraduate students at Russell Group universities.	Interview.	All users took Modafinil, with some also having tried Dextroamphetamine/amphetamine and Ritalin for the purpose of study. By recruiting both users and non-users, all non-users were found to be female.	All interviewees reported improving academic study as the primary purposes of drug use, particularly valuing improved focus, increased efficiency and reduced procrastination. These effects were judged to be highly desirable in the context of time constraints and fatigue when approaching exams and deadlines.
[33]	Iran	Modafinil, Methylphenidate, Amphetamine, Piracetam Vinpocetine	Vinpocetine.	Cross-sectional study was performed by analysing a total sample of 579 students in the one University of Medical Sciences students from 1st to 5th year.	Paper survey.	Some 44 (17.6%) of the respondents answered that they had used CEs at least once in their life, to increase concentration. There was a significant relationship between CE use and the age of respondents (*p* < 0.05). According to logistic regression analysis, there was a significant relationship between knowing someone who had used, stress level and CE use (*p* < 0.05).	Sample collection was one of the main limitations. For example, the female sample was larger than the male sample. Students entered the study without prior notice of it, which means that a factor may play a role, as well as memory bias, especially when students are being asked to record non-pharmaceutical use. Finally, it is recommended that a study should be conducted in all universities of Iran and their results compared. Therefore, although it is obvious that the use of these drugs for increasing cognition was investigated more among student populations, it is not possible to generalise to other populations.
[34]	Pakistan	Methylphenidate	-	A cross-sectional study was conducted in Medical colleges in Pakistan, using a self-constructed, validated questionnaire. The sample size (400) was calculated using open-source Statistics for Epidemio-logical Health software.	Paper survey.	Some 27 participants admitted the use of Methylphenidate to improve concentration. Peer pressure was found to be a major factor in its misuse.	The study determined the prevalence of non-therapeutic use of methylphenidate as well as ascertaining any benefits, side effects, and other factors associated with this use. This is a cross-sectional study and, apart from a chi-square test, no other statistical analysis could be performed. The study only includes two cities in Pakistan and must be expanded to include other regions as well, especially the regions labelled as high risk for drug misuse. This study does not extensively explore the reasons for a participant opting for drug abuse, regardless of academic performance or environment.
[35]	Brazil	Methylphenidate	-	Simple random sample of students of the Universidad Federal de Minas Gerais (*n* = 438), invited to answer an online questionnaire about the use of methylphenidate	Online survey.	Out of 378 students included, 5.8% (*n* = 22) reported using methylphenidate for CE; of them, 41% (9/22) in the 4 weeks prior to the survey.	The study estimates the prevalence of, and factors associated with, the use of methylphenidate for cognitive enhancement among undergraduate students.
[36]	Belgium	Methylphenidate, Amphetamine, Modafinil	-	A cross-sectional study of 3159 Medical students.	Paper survey and online survey.	Approximately 8.7% of the students reported that they used CE to improve their academic performance during exam time.	The study investigated the prevalence of the non-medical use of methylphenidate and knowledge of this drug among Undergraduate Medical students of the University of the Free State.
[37]	Canada	Methylphenidate, Amphetamine, Modafinil	Caffeine	11 focus groups, 3–7 participants per group.	Focus group interview.	Approximately 5% to 30% of students reported the use of CE.	The study has certain limitations. Firstly, for confidentiality reasons, they did not ask participants about their own history of using CEs. This precluded them from knowing when participants were truly referring to a friend in their narratives, or when they were following the interviewer’s instructions to mask their own illicit activities. Second, they did not directly question participants on how they knew about the effects of CEs, as this was an unexpected line of inquiry.
[38]	Italy	Coffee, Ginkgo-biloba, Caffeine, Amphetamine, Modafinil, Methylphenidate	Ginkgo biloba, Caffeine, Energy drinks	433 medical students.	Paper survey.	Approximately 74.7% of the students said they have used CE to improve cognitive functions. The remaining students were aware of concerns about safety and side effects.	The study explored the use and attitudes toward the use of CE in Italian Medical students. Only one university was involved; therefore, the generalizability of their findings to the whole Italian student population is limited.
[39]	Iran	Amphetamine, Methylphenidate	-	Cross-sectional study was conducted among 560 Medical students and clinical residents of Babol University of Medical Sciences during the academic year 2014–2015.	Paper survey.	Some 444 students (79.3%) filled out the questionnaires. 49 (11%) individuals reported amphetamine and methylphenidate (Ritalin) use. The mean age of the stimulant drug users was 24.6 ± 4.8 years. The main initiating factor was to improve concentration (29 persons; 59.2%).	The study was to evaluate the current situation of stimulant use among Medical students and residents of Babol University of Medical Sciences. The survey was conducted in class before the lecture started, so the students may have been in a rush to finish the questionnaires.
[40]	UK, France, Austria, Belgium, Brazil, Canada, German, Hungary, Ireland, New Zealand, Australia, USA, Portugal, Switzerland and the Netherlands	Methylphenidate, Modafinil, Amphetamine, Cannabis	-	(2015) *n* = 79,640 (2017) *n* = 29.758	Online survey. Non-probability sample. The Global Drug Survey is an annually conducted anonymous web survey on substance use. Two data sets from the male and female Global Drug Survey (GDS).	The Global Drug Survey (GDS) is the largest study on CEs drugs that has ever been conducted. Across both years, there were more male than female respondents. According to responses from both years, the main source of supply for CE drugs participants was the circle of friends (47.8%). One in ten indicated that the Internet was their main source (11.8%). Family members with a prescription (6.1%) and physicians (3.8%) were less common sources for stimulant drugs used for CEs. Overall, 4.9% and 13.7% of the global sample reported the use of CE drugs to improve performance at work or while studying.	Several limitations were considered: The first two and most important limitations of the study are the self-selection of GDS survey participants and the use of self-reported data. Since the sample is self-selected and the substance use for CE drugs consists of self-reported data, the actual extent of CE drugs in the participating countries is not accurately known. The sample should not be considered representative of any country’s general population. A third limitation is that the impact of recall bias or deliberate misreporting on results must be considered. Finally, due to the anonymous web survey instrument, the same individual might have completed the GDS2015 multiple times. However, <1% of the sample provided identical response sets across demographics and key variables used in these analyses.
[41]	Australia	Methylphenidate, Modafinil, Amphetamine	-	1136 Australian students.	Online survey.	6.5% reported that they used CE to improve academic performance.	The study found that the prevalence of non-medical prescription stimulant use, to improve academic performance, is low among university students in Australia. The cross-sectional design means that it is not possible to infer causal relationships between the use of prescription stimulants and other factors. The use of self-reporting measures may have introduced recall and social desirability biases.
[42]	Austria	-	Caffeine pills	2284 students.	Paper survey.	14.9% of participants reported the use of Caffeine pills.	(I) To investigate whether including caffeine tablets in the definition of pharmacological neuroenhancement (PN) within a questionnaire increases the PN prevalence estimate (framing effect), (II) To investigate whether the health-related risk attitude is increased in students who use PN.
[43]	Australia	Methylphenidate, Modafinil, Amphetamine	-	642 students.	Online survey.	6.32% of individuals reported lifetime use of one or more prescription CE drugs, listed for the purposes of study-related enhancement.	Even though this study provides some insights into the CE drug use that occurs at Australian universities, there are some limitations to consider. Results should be interpreted in the light of the convenience and cross-sectional sampling methods used. Participants studying Science degrees, women and undergraduates were also oversampled. As a result, the distribution of the students in the current study may not be an accurate representation of the entire student population at Australian universities. The number of illicit CEs drug users was also so low that statistical analyses were deemed inappropriate for this group. Therefore, caution should be exercised in interpreting the results, given the constraints of the sample. As per previous work, future studies may consider examining the academic outcomes of Australian students that use CE drugs, particularly contextualised regarding coping.
[44]	UK	Methylphenidate, Dextroamphetamine/amphetamine, Modafinil	Caffeine	All Level 1 and Level 4 M. Pharm.	Paper survey. Convenience sample.	The response rates were 89.3% (Level 1) and 89.0% (Level 4) with 48.0% of respondents reporting they were CE users (largely caffeine). Additionally, 42.4% thought using pharmaceutical CEs for improving academic grades breached their Code of Conduct.	The study could be done for other Schools, such as other healthcare disciplines. However, the opinions were captured at one point in time, data were self-reported, and the findings are not generalisable. Perhaps, if the study had been conducted immediately before the written examinations, prevalence of CE use would have been higher. Manually distributing paper-based questionnaires to students in a compulsory class and an online distribution would enhance the response rate. Other ways to maximise the response rate included having a relatively short questionnaire with questions largely as closed questions. Educational workshops could further explore ethical issues.
[45]	Greece	Amphetamine, methylphenidate, Cannabis	-	591 Medical students.	Online survey.	About 1 in 10 medical students misused prescription drugs, mostly for self-treatment purposes and about 1 in 4 used illicit drugs, mostly for recreational purposes, with cannabis being the most frequently used.	To analyse the prevalence of lifetime and current use of illicit drugs among Medical students worldwide. Considering that CE use during medical school affects students’ personal and professional lives, further international studies are needed to elucidate the prevalence and the motivation of that use among medical students.
[46]	New Zealand	Methylphenidate, amphetamine, Modafinil	-	449 Pharmacy, Medicine, Nursing and Law Students.	Paper survey.	Response rate was 88.6% (442/499). Prevalence rate of CE was 6.6% in the university environment sampled There were no significant differences in student motivation and learning strategies between users of CE and non-users.	To investigate what factors explain the decision to use CEs among tertiary students in New Zealand, using the Theory of Planed Behaviour. This research supports the notion that the decision to use CEs is not just an autonomous choice that occurs in isolation. Attitudes on the ethical and social acceptability of CE use were more likely to drive the decision to use CEs.
[47]	Greece	Methylphenidate, Modafinil	ginseng, taurine, caffeine, Vitamin B complex	450 university students.	Paper survey.	The findings show that university students may engage in pharmacological cognitive enhancers’ (PCE) use independent of their student experiences. Rather, a chemically assisted performance enhancement mindset seems to differentiate users from non-users of PCEs.	The study did not address whether such achievement motivations underlie the decision-making processes to use PCEs among university students.
[48]	South Africa	Methylphenidate	-	Year 5 Undergraduate Medical students (541 students).	Paper survey.	Some 11% reported the use of methylphenidate for study enhancement purposes.	There are few limitations in this study. The survey was self-administered, and the questionnaire was conducted in class before the lecture started, so the students may have been in a rush to finish it. The questionnaire was not structured in such a way to determine whether participants with Attention Deficit Hyperactivity Disorder (ADHD) were using methylphenidate as prescribed or misusing it for reasons not related to their ADHD.
[49]	UK	Methylphenidate, Amphetamine, Modafinil	-	66 participants from Russell Group universities.	Focus group interview.	Some 58/66 students thought that it is a good choice to use CEs.	This study was conducted to compare the acute effects of methylphenidate/MPH, modafinil, and 3,4-methylenedioxymethamphetamine on the neural mechanisms underlying response. Not able to disentangle neural activation in response to successful vs. failed inhibitions in the present study due to the modest number of no-go trials. The small number of inhibition trials (i.e., no-go trials) also limited the functional relevance of the behavioural results, albeit MPH and modafinil significantly increased the probability of inhibition.
[50]	Australia	Methylphenidate, Amphetamine, Modafinil	-	38 students.	Interview.	*n*= 5 had used CEs.	CEs users reported higher levels of stress and lower levels of ability to cope than the sample average.
[51]	United Arab of Emirates	-	Caffeine	175 university students in one university only: Year 1 to year 4. The Schools of: Art and Creative Enterprises. Business Sciences, Communication and Media Sciences. School of Education and School of Sustainability Sciences	Paper survey. Convenience sample.	Eighty-six per cent of the 175 participants, both males and females, at Zayed University, Dubai consumed caffeinated beverages with an average intake of 249.7 ± 235.9 mg. The intake among the 150 caffeine consumers varied from 4.2 mg/day to 932.2 mg/day.	The study was to determine the prevalence of caffeinated beverage consumption among university students and the perceived benefits. In addition to the estimation of daily caffeine consumption, the study was undertaken in one university only. In the UAE, the limited studies that were done regarding this concern showed the high tendency of university students towards the consumption of caffeinated drinks, mainly energy drinks.
[52]	Iceland	Methylphenidate, Dextroamphetamine/amphetamine, Modafinil	-	*n* = 521.	Online survey.	Approximately 11% used CE without prescription, 42% had a prescription. The reason for the misuse was to improve their academic performance.	To review historical information concerning prescription stimulants and to summarise the literature with respect to misuse among adults, particularly college students, including risk factors, mediators and moderators, and motivations for prescription stimulant misuse. Lack of understanding in variability according to dose level and individual variability is a clear limitation across most studies examining the potential for neurocognitive enhancement from prescription drugs.
[53]	France	Methylphenidate, Modafinil, 3,4-Methylenedioxymethamphetamine (MDMA or ecstasy), piracetam, amphetamine.	-	1718 Medical students and physicians.	Online survey.	Approximately 33% reported the use of CE, mainly to increase academic performances and to stay awake during exam preparations.	To estimate the prevalence of psychostimulant use in the French medical community and their motives. Lack of direct information on the period of stimulant use. It was a choice to keep the questionnaire short to maximize the response rate. This limit was partially addressed by the age at the first psychostimulant use, which was considered in the analyses.
[54]	Lithuania	Modafinil, Methylphenidate, Amphetamine	-	A cross-sectional survey study was performed by analysing a convenience sample of *n* = 579 in the two universities offering Medical education in Lithuania.	Paper survey.	Approximately 8.1% reported that they had used CE in their lifetime.	To analyse the use of cognitive enhancers among medical students in Lithuania, to determine the reasons for usage and evaluate the contributing factors, such as sociodemographic characteristics, stress levels, sleep quality and knowing somebody who has used a neuro-enhancing drug. Students participated in the study without any previous knowledge about it, which means that a surprise factor may have played a role and memories could be biased.
[55]	Lithuania and the Netherlands	Racetam group substances Benzodiazepines Modafinil, Methylphenidate, Amphetamine	Caffeine pills	Interview *n* = 35 Survey *n* = 113	Online survey and Interview	From 113 respondents in the survey, 24 (21%) reported having tried CEs. Most of participants turned to CEs to enhance their concentration for the purpose of study and time management.	Future research needs to take into account the great variety of drugs/substances that students use as CEs in real-life settings.
[56]	The Netherlands	Methylphenidate, Modafinil, Rivastigmine, Beta Blockers	-	1572 students.	Online survey.	No response was reported on the use of Modafinil and Rivastigmine. 52 students reported the use of methylphenidate. 36% had used Beta Blockers at least once in their lifetime.	Convenience sampling constituted only an approximate representation of the student population in the Netherlands. Women, for example, were oversampled. In addition, the sample was not equally distributed for different universities, as well as not distributed being in line with the absolute difference in number of students of the 14 Dutch Government supported universities.
[57]	Iran	Methylphenidate	Alcohol	16,000 medical students.	Paper survey.	The prevalence of prescription drug misuse, alcohol use in the previous year, and every illicit substance use was 4.9%, 6.9%, and 2.9%, respectively.	There is limited information about illicit drug use and associated factors in hookah smokers in Iran. So, the aim of this study was to assess the status of illicit drug use and associated factors among hookah smokers of Khalil Abad city in 2015.
[21]	Switzerland	Methylphenidate, Modafinil	-	Students at three Swiss universities were invited by email to participate in a web-based survey. Of the 29,282 students who were contacted, 3056 participated.	Online survey.	Approximately 22% used CE to improve cognitive performances while studying.	Investigate students’ attitudes toward PCE. The response rate for the present survey was 10%. The study sample may not have been necessarily representative of all Swiss students. Although all students from UniBas and ETHZ (ETH Zürich University) were invited, only 5000 of a total of 26,000 students who were currently enrolled at University of Zürich (UZH), who had previously agreed to be contacted for participation in various studies, could be invited.
[22]	UK and Ireland	Modafinil, Methylphenidate, Dextroamphetamine/amphetamine	-	877 students in 104 universities.	Paper survey of a convenience sample of 877 students measured PCE prevalence, attitudes, sources, purposes and ethics.	Only 2% reported that they have used CE.	Results from the convenience sample survey may be biased, due to participants’ self-selection. They only used an online survey, which was considered too costly and unfeasible due to access barriers.
[58]	Northern Ireland, Wales and England	Cannabis, Ecstasy, amphetamines	-	3706 students from 7 universities across Northern Ireland, Wales and England. England (Gloucestershire *n* = 908, Bath Spa *n* = 462, Oxford Brookes *n* = 203, Chester *n* = 883, Plymouth *n* = 167); Wales (Swansea *n* = 398); and the Republic of Northern Ireland (Ulster *n* = 463). Each participating institution provided ethical approval.	Paper survey. Convenience sample.	Some 5% reported that they had regular use of CE, and 25% used CE occasionally, and 70% never.	The study could be carried out at other schools, such as other in healthcare disciplines. However, the opinions were captured at one point in time, data were self-reported, and the findings are not generalisable. Perhaps if the study had been conducted immediately before the written examinations, prevalence of CE use would have been higher. Manually distributing paper-based questionnaires to students in a compulsory class and an online distribution would enhance the response rate. Other ways to maximise the response rate included having a relatively short questionnaire with questions largely as closed questions.
[59]	Switzerland	Methylphenidate, Dextroamphetamine/amphetamine, Modafinil	-	1765 students.	Online and paper survey.	4.7% had used CE for the purpose of studying.	The findings from this survey can lead to a better understanding of why some students are already using CE and can also add to the discussion on social norms and values in the context of legalizing or prohibiting such products.
[60]	France	Methylphenidate Modafinil, amphetamines, Piracetam	-	206 students.	Online survey sent to a French sample of Medicine and Pharmacology students using email.	Among 206 undergraduate students, 139 students (67.4%) declared to have consumed at least one cognitive enhancer in the past 12 months. Twelve students (8.6% of cognitive enhancers users and 5.8% of our total sample) used illicit pharmaceutical neuroenhancers.	Assess prevalence and motivations for licit (use inside medical indication) and illicit pharmaceutical neuroenhancer consumption (tablet form) in a non-selected French sample of Medicine and Pharmacology students. A prevalence of 5.8% for smart drugs consumption in Pharmacology and Medical students, mostly in order to enhance academic performances and vigilance was recorded. Methylphenidate was the most frequently consumed molecule.
[61]	USA	Methylphenidate, Dextroamphetamine/amphetamine	-	4 years repeated study.	Online survey.	1 in 5 students reported the use of CE at least once in their lifetime.	Examined stimulants’ cognitive enhancement effects and the psychological profile of non-medical stimulant users. A double-blind, placebo-controlled experiment found no enhancing effect of mixed amphetamine salts (Adderall) on healthy participants’ inhibitory control, working memory, episodic memory, convergent creativity, perceptual intelligence and a standardized achievement test. No moderating effects of baseline performance or Catechol-O-methyltransferase (COMT) genotype were detected.
[62]	Germany	Amphetamine, Methylphenidate, Ecstasy, Cocaine	-	18 participants.	Interview.	Among all participants (*n* = 18 = 100%), 77.8% (*n* = 14) had used illicit stimulants (AMPH) and 38.9% (*n* = 8) prescription stimulants (MPH). 22.2% (*n* = 4) had used prescription as well as illicit stimulants for academic performance enhancement.	Several limitations were reported. One of them is the limited number of interviews: Only 18 interviews were taken into consideration. In spite of the fact that the University population was 36,000 registered students who had the possibility to notice the advertising placards of this interview study throughout the campus, only 30 students contacted them, and only 22 were willing to participate. Given CE prevalence rates of 3–20%, there should have been a much higher number of potential participants for this study. They hypothesize that the stigmatizing subject of this study is the reason for the low participation rate, notwithstanding the fact that anonymity was guaranteed and that participants were remunerated for their time and effort with 30 Euros.
[63]	USA	Methylphenidate, Amphetamine	-	1115 medical students a multi-institutional census using a 31– 48 item online survey regarding use of prescription psychostimulants	Online survey.	Approximately 18% had reported that they used CE at least once in their lifetime.	Given that students’ responses are self-reported, and that non-medically prescribed stimulant use is illegal, misreporting is a potential concern in this survey. However, the survey did not distinguish between giving away (illegal) or selling (criminal) these drugs. Previous studies have indicated that anonymous self-reported surveys have low misreporting rates.
[64]	USA	Dextroamphetamine/amphetamine (Adderall)	-	213,633 tweets.	Online survey.	Approximately 12.9% tweets concerned the use of Adderall for studying purposes.	First, not every Adderall tweet was related to actual use. For example, they observed song lyrics that impact these counts, such as the two often quoted lines “College hoes love alcohol and popping Adderall” and “I’ve been up for 3 days… Adderall and red bull.” In our sample, there were 4275 tweets that have the words “college hoes love” and 894 that have the words “been up for three 3 days”. These numbers probably inflate the number of matches for “college”, “alcohol”, and “red bull” above the number of people tweeting about using these substances.
[65]	Germany	Dextroamphetamine/amphetamine, Modafinil, Methylphenidate	-	2569 students.	Paper survey.	An estimated 12-month prevalence of using cognitive enhancing drugs was 20%. Prevalence varied by sex (male 23.7%, female 17.0%), field of study (highest in students studying Sports-related fields, 25.4%), and semester (first semester 24.3%, beyond first semester 16.7%).	As a result of the study findings, drug prevention models need to be established at all universities in Germany.
[66]	USA	Dextroamphetamine/amphetamine, Methylphenidate, Modafinil	-	372 Medical, Pharmacy and Respiratory Therapy students.	Online survey.	Approximately 10.9% Medicine, 9.7% Pharmacy and 26.3% Respiratory the students reported the use of CE to enhance alertness and improve academic performance.	The incidence of psychosis or withdrawal associated depression is not known for prescription drugs.
[67]	Canada	Methylphenidate, Modafinil	Caffeine pills	647 Medical students across all four years.	Online survey.	Approximately 8% of the Seniors report the use of CE vs. 2% of Junior students using CE for cognitive enhancement.	It was carried out at a single institution; however, we have no reason to believe that the results are not generalizable to students studying elsewhere. While self-selection may have led to a positive response bias, it is equally plausible that non respondents did not wish to disclose use of cognitive enhancers.
[68]	UK	Methylphenidate	Caffeine pills	1614 students.	Online survey.	Approximately 33% had used drugs without prescription of which 0.5% used stimulants for a studying reason. 6% used caffeine pills.	The limitation in this study is that the response rates are quite low and also the study is exposed to the limitations of all self-reported surveys.
[69]	Italy	Modafinil, Methylphenidate, Dextroamphetamine/amphetamine	-	77 Undergraduate students.	Paper survey.	Approximately 16% reported they had taken CE in the past.	The limitation in this study is the question on CE use which did not specify what exactly the students took; their behaviour risk is difficult to assess and assumes that the truly problematic behaviour is to take CE drugs without having a prescription.
[70]	Iran	Methylphenidate	-	Group of Medical students	Paper survey.	Approximately 8.7% reported the use of methylphenidate at least once in their lifetime.	The first limitation is the validity of self-reported methylphenidate use among respondents which depends on their willingness to reply truthfully to the survey. Second, the sample in the study was from one university, thereby necessitating that similar studies be conducted in other medical schools for comparison. Third, the study did not explicitly address duration or frequency of methylphenidate use. Therefore, it is unknown whether non-prescription users took methylphenidate regularly or only occasionally.
[71]	Germany	Methylphenidate, Dextroamphetamine/amphetamine, Modafinil, MDMA	-	1035 students of pupils from (Vocational and Grammar Schools) and 512 students from Medicine, Pharmacy and Economics Schools.	Paper survey.	Approximately 1.55% of pupils from Vocational and Grammar School vs. 0.78% among students in Medical, Pharmacy and Economics reported a lifetime provenance for CE use. 2.42% of pupils vs. 2.93% of students reported lifetime use of CE for cognitive enhancement.	Data sampling was non-random, participants were not able to refuse participation in a discrete way. At least in the student population, in which approximately 30% did not fill in the questionnaires; it cannot be excluded that stimulant use is underreported since especially students with “negative behaviours” did not fill in the questionnaires, leading to underreporting of stimulant use.
[72]	USA	Dextroamphetamine/amphetamine, Methylphenidate	-	4580 students.	Online survey.	Approximately 75.8% reported that they have used amphetamine (Adderall) in the past year, 24.5% used methylphenidate (Ritalin).	Sample consisted of students from a single university, which may limit the generalizability of the results. However, the prevalence rates of illicit use of prescription stimulants in this single institution study were comparable to those found in national surveys of college students.
[73]	USA	Dextroamphetamine/amphetamine, Methylphenidate	-	9161 students.	Paper survey.	Approximately 8.1% reported lifetime use of CE, 5.4% reported past year use of CE to increase alertness and concentrate better.	The 2001 College Alcohol Survey (CAS) did not measure legitimate medical use of prescription stimulants or diagnosis, so it was not possible to assess how many students with legitimate prescriptions for stimulants may have misused their own or someone else’s stimulant medication. As the data were cross-sectional, inferences about causality are limited and they could not assess whether certain factors preceded initiation of non-medical use of prescription stimulants. Longitudinal data are needed to further examine the directionality of these associations.
[74]	USA	Methylphenidate, Dextroamphetamine/amphetamine, MDMA	-	150 students.	Paper survey.	Approximately 35.3% reported they had misused Amphetamine once in their lifetime, 10% abused it monthly and 8% weekly.	Reports of stimulant use are high in the research; it may be that a relatively small sample was not representative of college students in general, despite attempts to avoid selection bias. A significant proportion of students came from Undergraduate Psychology classes and these students may differ from those in other Majors. Alternatively, it is possible that small, competitive colleges attract students who have been exposed to stimulant use, or who are willing to experiment with Amphetamines to enhance academic performance.

**Table 2:** Studies summarising Cognitive Enhancers (CEs)’ legal classification, desired effects and neuro-modulatory mechanisms.

Drug/Substance	Brand Name	Misuse of Drugs Regulation (2001) (UK)	Misuse of Drugs Act 1971 (UK)	Currently Recommended Clinical Use and Neuro-Modulatory Mechanism
Amphetamine salts	Adderall	Schedule 2	Class B	Amphetamines are a class of pharmaceuticals that include Adderall, dextroamphetamine, and lisdexapmhetamine (L-lysine-d-amphetamine) [91]. These drugs were developed to treat attention deficit hyperactivity disorder (ADHD) in adults and children [4]. These molecules are classified as Schedule II according to the Misuse of Drugs Regulation (2001) and Class B according to the Misuse of Drugs Act 1971, due to their high abuse potential. Even though the risk of developing dependence on these drugs is believed to be low for individuals taking them for ADHD, the Schedule II classification indicates that there is a high potential for abuse and severe dependence [4]. These drugs were also demonstrated to improve episodic memory, working memory, and some aspects of attention in general population [92]. The therapeutic effect of both amphetamine and methylphenidate in ADHD is consistent with the finding of the abnormalities in the catecholamine system in individuals with ADHD [93,94].
Caffeine	Genius Caffeine	Over-the counter (OTC)	-	The usage of caffeine is increasing worldwide [95]. The underlying motivations are mainly memory and concentration enhancement and physical performance improvement. Coffee and caffeine-containing products affect the central nervous system, with their locomotor activity stimulation and anxiogenic-like effects [78]. Caffeine also impacts on other neurotransmitters, including dopamine, noradrenaline, serotonin, glutamate, acetylcholine and gamma-aminobutyric acid [96]. Caffeine consumption is very prevalent among the UK [97] and UAE [51] population. Healthy consumption needs to be promoted [51]. Although caffeine is also a stimulant, it is not illegal to use without a prescription [66].
Cyanocobalamin (vitamin B12)	Athlete	OTC	-	It may help patients on long-term medications and those with neurological disorders [98]. Cognitive performance can be improved, and the risk of brain atrophy reduced, by Vitamin B12 [99].
Guarana (Paullinia cupana)	N-R-G	OTC	-	*Paullinia cupana* is a plant native to the Amazon basin which is especially common in Brazil [100]. A review study on the effect of Guarana among healthy individuals reported an improvement in reaction time and accuracy of performance at cognitive tasks [82]. Guarana seeds are popular worldwide for their cognitive, stimulant and behavioural effects [82].
Methylphenidate	Ritalin	Schedule 2	Class B	It is a stimulant drug used to treat ADHD and narcolepsy. It has been controlled as Schedule II according to the Misuse of Drugs Regulation (2001) and Class B according to the Misuse of Drugs Act 1971 due to its high abuse potential. Volkow and colleagues (2004) showed the effects of methylphenidate on motivation, which can affect academic performance whilst increasing cognitive ability and improving students’ self-rated interest in a relatively dull mathematical task. A study reported that methylphenidate has one of the highest prescriptions rates, associated with an abundance of websites offering to sell and supply the drug without a prescription to UK users [30]. University students might be attracted to methylphenidate because of its alleged increase in attention and focusing levels [101]. Among university students, the self-reported misusing rates were from 1.5 to 31% depending on the different surveys considered, with the most nationally (German white males, affiliated with a formally organised fraternity) representative study estimating an annual illicit methylphenidate usage of about 4% [101].
Modafinil	Provigil	Prescription-only-medicine (POM)	-	Wakefulness-promoting agents such as modafinil and armodafinil are stimulant drugs which are used in the treatment of narcolepsy and shift workers sleep disorders [30]. The mechanism of action of modafinil is poorly explained in the literature. It has been reported that modafinil affects GABAergic and dopaminergic pathways in the prefrontal cortex and has effects on neurotransmitter systems (e.g., noradrenaline and dopamine) [80]. Modafinil is praised for its ability to improve reaction time, logical reasoning and problem solving [77].
Piracetam	Nootropil	POM	-	Compounds from the racetam family include piracetam, oxiracetam, etc [102]. Piracetam belongs to the nootropic drugs’ group which includes the brain cell metabolism and energy enhancement [103]. Although Piracetam is officially recognized as a nootropic, its enhancing effects in the healthy individual’s brain are moderate [104,54]. The racetam molecules are being used across a range of brain disorders, including Alzheimer’s disease, narcolepsy, ADHD, Parkinson’s disease and brain aging [105,106].
Pyridoxine (vitamin B6)	Nestrex	OTC	-	Pyridoxine, one of the most common forms of Vitamin B6 [107], is said to significantly improve verbal memory and executive function [108]. It can aid in the synthesis of neurotransmitters and amino acids. Some of these neurotransmitters are norepinephrine, serotonin, GABA and dopamine [108]. There is no evidence that Vitamin B6 short-term use (e.g., for 5–12 weeks) improves cognitive function or mood [109]. More evidence is needed to determine whether Vitamin B6 supplements might improve cognition in healthy people.
Vinpocetine (Vinca minor)	Cavinton	OTC	-	Is an alkaloid of the periwinkle plant (*Vinca minor*) [110], which has been shown to exert a brain neuroprotective effect by a combined action on brain metabolism, cerebral circulation and rheological properties of the blood. This may boost the cerebral metabolism thus enhancing both oxygen and glucose utilization whilst consequently improving cerebral functions and providing protection even in conditions of hypoxia and ischaemia [111]. It is commonly used as a nootropic that promotes memory formation [106].
